# The Utility of Cognitive Screening in Asian Patients With Heart Failure: A Systematic Review

**DOI:** 10.3389/fpsyt.2022.930121

**Published:** 2022-07-14

**Authors:** Qi Niu, WeiHua Liu, FengLing Wang, LiYa Tian, YanHong Dong

**Affiliations:** ^1^School of Nursing, Shandong First Medical University & Shandong Academy of Medical Sciences, Taian, China; ^2^Linyi People's Hospital, Linyi, China; ^3^Alice Lee Centre for Nursing Studies, Yong Loo Lin School of Medicine, National University of Singapore, Singapore, Singapore

**Keywords:** heart failure, cognitive screening, utility, Asia, systematic review

## Abstract

**Background:**

The prevalence of undiagnosed cognitive impairment in patients with heart failure is alarmingly high in Asia. There is still no consensus on cognitive screening tools to detect cognitive impairment in the Asian heart failure population. The clinical implications based on our systematic review may help to improve cognitive screening practice for patients with heart failure in Asia.

**Methods:**

This review is registered in the PROSPERO (CRD42021264288). Using the Preferred Reporting Items for Systematic Reviews and Meta-Analyses (PRISMA) approach, we searched PubMed, Embase, the Cumulative Index to Nursing and Allied Health Literature, Scopus, the Web of Science, PsycINFO, the Cochrane Central Register of Controlled Trials, China National Knowledge Infrastructure, and Wanfang Data in English and Chinese literatures concerning heart failure and cognitive impairment.

**Results:**

The search yielded 21 eligible studies. Only in five studies, cognitive brief tests, including the Montreal Cognitive Assessment (MoCA), the Mini-Mental State Examination (MMSE), and the Mini-Cog, were used as cognitive screening tools for Asian patients with heart failure. In the rest 16 studies, brief cognitive tests were used as screening tools for global cognition. Only one study validated screening tests against a gold standard formal neuropsychological assessment test battery. Among these studies, patients with heart failure tended to perform worse than patients without heart failure. The presence of cognitive impairment in patients with heart failure is associated with poorer self-care, quality of life, and hospital readmission.

**Conclusion:**

Brief cognitive tests have been used in Asian patients with heart failure and these tests are frequently used as a measure of global cognitive function for cognitive screening. However, validating brief cognitive tests against a gold standard formal neuropsychological assessment in Asian patients with heart failure is lacking. Future studies need to address methodological issues to validate cognitive screening measures in a larger population of Asian patients with heart failure.

**Systematic Review Registration:** https://www.crd.york.ac.uk/prospero/

## Introduction

Heart failure (HF) is a clinical syndrome resulting from any structural or functional cardiac disorder that impairs the ability of the ventricle to fill or eject blood (1). HF is a rising global health epidemic affecting approximately 63.4 million people worldwide and 80% of patients with heart failure are 65 years or older. The prevalence of heart failure increased exponentially with age (2); the incidence rate of HF population under 55 years is 1%, while the incidence rate is over 10% for HF people aged 70 years or above. It is one of the leading causes of hospitalization, morbidity, and mortality in Asian countries, with an incidence that ranges from 1.2 to 6.7% (3). In particular, China has an estimated 13.7 million individuals with HF (4); among them, elderly patients account for 75%. About 2–17% of individuals admitted to a hospital with HF die while in a hospital (5). Patients with HF have a readmission rate of 25% within 30 days of initial discharge (6). Moreover, 17–45% of patients with HF die within 1 year of hospital readmission, while 50–80% HF population die within 5 years of admission (5). This considerably increases healthcare costs at both the individual and societal levels.

Studies have shown that HF is highly correlated with cognitive impairment (CI) (7), leading to poor health outcomes such as poor self-care and medication compliance. HF is associated with significant risk (>80%) of developing dementia and Alzheimer's disease (8). The prevalence of CI is as high as 25–80% in the HF population worldwide, with the prevalence of CI among older and hospital patients higher than in community-dwelling patients (9–13). Cognitive functioning includes various abilities and skills such as memory, attention, and executive function (e.g., planning, organization, and problem-solving) and is central for patients with HF to carry out their activities of daily living (14). Patients with HF may lose short-term memory and have difficulty with concentration (15, 16). resulting in difficulty in medication compliance and other self-care activities. This, in turn, leads to a higher rate of readmission and increased mortality (17, 18).

A recent study indicated that CI in Asian patients with HF is alarmingly high, i.e., 44% (19). Additionally, CI is closely associated with poor prognoses, such as suboptimal treatment adherence and self-care, hospital readmission, and increased mortality (20, 21). Early detection of CI is, therefore, an important step to achieving early intervention and customized care for Asian patients with HF.

Although cognitive screening in HF is a pressing need, there is still no consensus on cognitive screening tools to detect CI in the Asian HF population. Some studies determined the cognitive function of patients with HF by a formal neuropsychological assessment (10). Although a formal neuropsychological assessment is the gold standard to establish CI, yet, they are lengthy, costly, and difficult to be implemented in routine clinical practice. By comparison, brief cognitive screening tools such as the Mini-Mental State Examination (MMSE) and the Montreal Cognitive Assessment (MoCA) were practical and frequently adopted in various HF studies. While the MMSE has been the most frequently used screening tool in HF research, its drawback is poor sensitivity. The comparative study of the MoCA appears to be a more promising screening tool to detect CI in HF (22).

Cameron and their colleagues (23) conducted a systematic review of studies from January 1999 to June 2013 to determine the diagnostic accuracy of cognitive screening tools in detecting CI in patients with HF and indicated that the MMSE had low sensitivity (26%) and high specificity (95%). Subsequently, Davis and their colleagues (22) reviewed literature published from January 2000 to May 2011 to evaluate cognitive screening tools and determine their usefulness and feasibility in clinical practice. They found that the MMSE did not detect CI in the domains frequently impaired in patients with HF. The MoCA was found to be a suitable screening tool for patients with HF (22). Both the systematic reviews have highlighted the critical need to examine the utility of cognitive screening in patients with HF and establish more suitable screening measures (22, 23). However, the conclusion of these two reviews was largely based on studies of the Western HF population. So far, there has been no review to systematically evaluate the utility of cognitive screening in Asian patients with HF. Therefore, our systematic review aims to examine the utility of cognitive screening in Asian patients with HF. The clinical implications based on our systematic review may improve cognitive screening practice, diagnosis, and management of CI among Asian patients with HF (24).

## Methods

This systematic review is reported following the Preferred Reporting Items for Systematic Reviews and Meta-Analyses (PRISMA). It includes cross-sectional, case–control, and longitudinal studies on the utility of cognitive screening in Asian patients with HF. This review is registered in the PROSPERO (CRD42021264288).

### Search Strategy and Study Selection

The following electronic databases were searched from inception in June 1984 up to June 2020: PubMed, Embase, the Cumulative Index to Nursing and Allied Health Literature (CINAHL), Scopus, the Web of Science, PsycINFO, the Cochrane Central Register of Controlled Trials, China National Knowledge Infrastructure (CNKI), and Wanfang Data. Terms used in the search strategy, include “HF,” “CHF,” “heart failure,” “cardiac failure,” “cognitive impairment,” “cognitive disorder,” “cognitive decline,” “cognitive dysfunction,” “cognition,” “dementia,” “MCI,” “Alzheimer's disease,” and “Asia (or individual Asian counties),” were used to search English and Chinese literatures concerning HF and CI. We combined the search terms using Boolean operators “AND” and “OR” (25–27). Database limitations included age 18 years or older, published as full studies in English and Chinese, and full text of original research. All the non-primary study literatures were excluded, such as literature reviews, dissertations, theses, editorials, protocol studies, and clinical guidelines (28). The search strategy and study selection were conducted independently by two reviewers (QN and LYT) with a consensus reached among these two reviewers.

### Data Extraction and Quality Assessment

Two reviewers (QN and LYT) screened titles and abstracts of all the articles from the databases and extracted data independently to prevent bias. Discrepant views were discussed and decided with the third reviewer (YD). If the study lacks sufficient information, its author was contacted to obtain relevant information. The following data were extracted:

- Identification of the study (first author; publication year).
- Methodological characteristics [study objective; sample characteristics (e.g., sample size, age, study region); heart failure criteria; cognitive screening tools; cutoff values; a measure of cognitive severity; sensitivity; specificity; positive predictive value (PPV); and negative predictive value (NPV)].
- Main findings and implications for clinical application.

The quality of the studies was assessed using a combination of two bias risk tools: the Quality Assessment of Diagnostic Accuracy Studies 2 (QUADAS-2) (29) tool and the Standards for Reporting of Diagnostic Accuracy (STARD) (30). The QUADAS-2 is developed to assess the quality of primary diagnostic accuracy studies and should be applied in addition to extracting primary data (such as study design and result), which is assessed as “yes,” “no,” or “unclear.” The purpose of the STARD initiative is to improve the quality of the reporting of diagnostic studies (31). The STARD checklist has 25 items, including study aims, participant sampling, data collection, demographic characteristics, and so on. The items in the checklist can help authors in describing essential elements of the design and conduct of the study, the execution of tests, and the results (31). Both the tools are used to assess the potential for bias and evaluate the generalizability of the results; hence, the use of these two tests is appropriate for assessing the quality of the studies reviewed. Disagreements of the quality assessment will be resolved by the third reviewer (YD).

### Data Synthesis and Analysis

The diagnostic criteria for neuropsychological impairment are met if there is a significant and evident decline in 1 or more cognitive domains (32). Cognitive decline is based on: (1) a concern about the individual's cognitive abilities and (2) performance on a battery of neuropsychological tests that is equal to or greater than 1.5 SD less than the age and education standardized means (33). We used published criteria whereby cognitive performance is standardized against an appropriate comparison group and impairment was operationalized as falling 1.5 SDs less than an appropriate comparison. As such, there was only one study that reported the diagnostic test accuracy of the MMSE and the MoCA and we were unable to examine pooled estimates of its accuracy from other publications. Moreover, most studies showed considerable methodological differences (i.e., sample size, time points of cognitive testing, education level, age group of patients with HF, cardiac function classification). Therefore, the results were too heterogeneous and, hence, not suitable for meta-analysis. Summaries of findings are tabulated to inform a narrative synthesis of the included studies.

### Patient and Public Involvement

As this is a systematic review, the patients and public are not directly involved in the design and development of this study.

## Results

### Search Results

**Figure 1:**
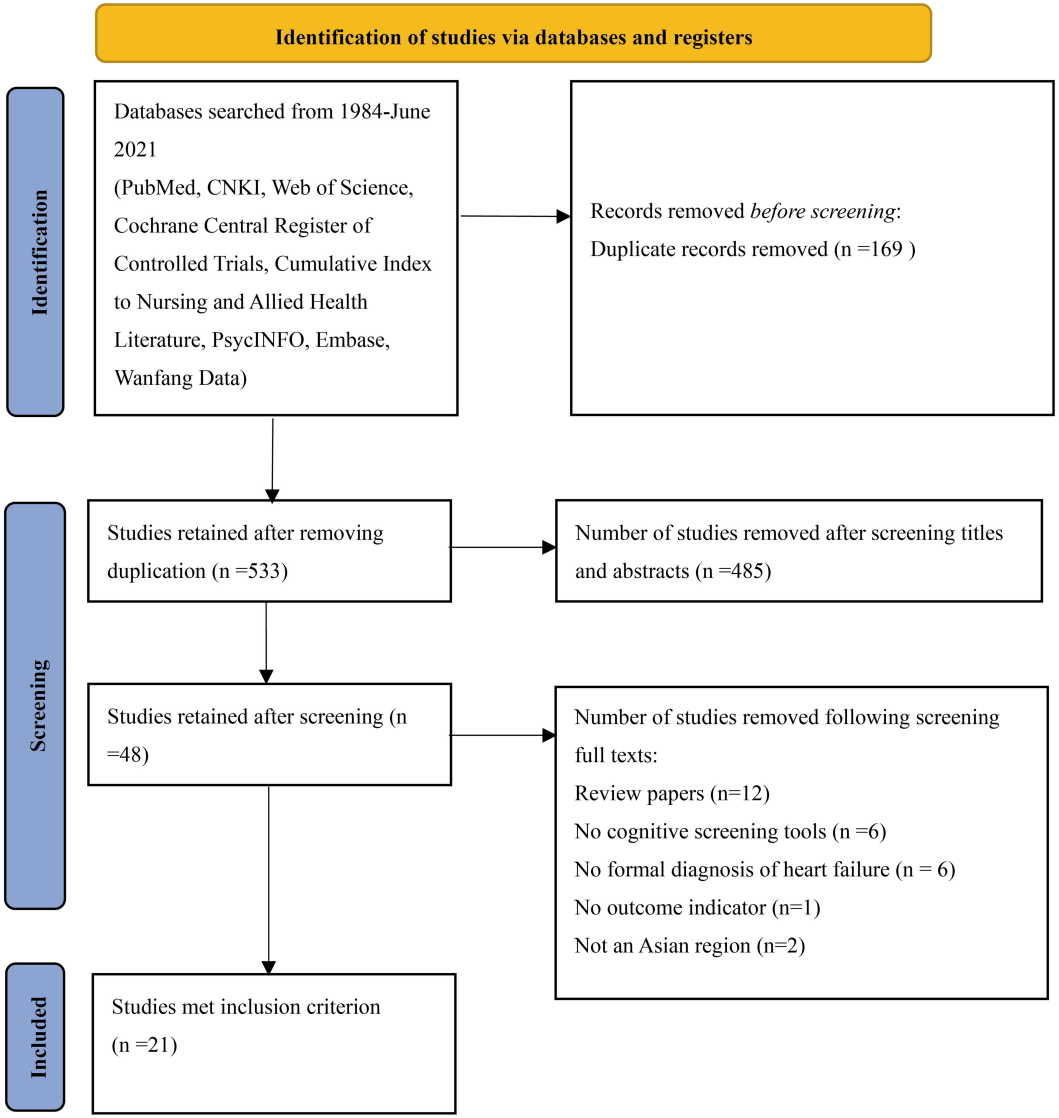
PRISMA 2020 flow diagram.

The database search yielded 702 studies (the CNKI 36, the Web of Science 61, PubMed 50, Wanfang Data 130, the Cochrane Central Register of Controlled Trials 24, Scopus 284, Embase 114, PsycINFO 1, and the Cumulative Index to Nursing and Allied Health Literature 2). Using EndNote 20, 169 duplicates were removed. Subsequently, 485 studies were removed after screening the titles and abstracts. A total of 27 studies were further removed after screening full-text articles (12 review articles, 6 without cognitive screening tools, 6 without a formal diagnosis of HF, 1 without an outcome indicator, and 2 not in Asian regions). Finally, 21 studies were eligible for inclusion (Figure 1).

### Characteristics of Included Studies

Most studies were cross-sectional studies (*n* = 7) (34–40), case–control studies (*n* = 10) (41–50), while the remaining studies were longitudinal studies (*n* = 4) (19, 51–53). Tables 1, 2 summarize the main characteristics of the 21 included studies. Studies were conducted in Asian regions, including China (*n* = 16) (19, 37–50, 52), Japan (*n* = 3) (34, 35, 51), Korea (*n* = 1) (36), and Singapore (*n* = 1) (19). Nine of the 21 included studies (19, 34, 35, 37, 40, 42, 50–52) used the MMSE as a cognitive screening tool, while there were 14 studies (19, 36, 38, 39, 41, 43–49, 52, 53) used the MoCA and 1 study (51) used the Mini-Cog.

**Table 1:** Comparison of cognitive screening instruments used in patients with heart failure.

**Study**	**Region**	**Participant Characteristics**	**Screening Measure**	**Screening Cut-Off**	**Measure of Cognitive Severity**	**SE,% (CI)**	**SP,% (CI)**	**AUC**
Bateman et al. (34)	Japan	*n* = 1,611 HF (criteria for HF was not specified)	MMSE	28		71	41	0.58
Dong et al. (19)	Singapore	ESC criteria *n =* 100HF age:58.68 ± 10.53	MMSE MoCA	MMSE <28 MoCA <25	A comprehensive formal neuropsychological test battery	79/71	63/61	0.740(0.641–0.840)/0.770(0.675–0.866)
Saito et al. (51)	Japan	Framingham criteria *n =* 352HF age:83 ± 5	MMSE Mini-Cog	MMSE <24 Mini-Cog ≤ 2				0.59(0.51–0.66)/0.52(0.43–0.60)
Saito et al. (35)	Japan	Framingham criteria *n =* 184HF age:82 ± 7.2	MMSE	24				
Yanqiu et al. (53)	China	NYHA *n =* 50HF age:64.2 ± 9.6	MMSE MoCA	MMSE <26 MoCA <26				

**Table 2:** Summary of publications meeting the inclusion criteria of this systematic review.

**Study**	**Region**	**Participant characteristics**	**HF criteria/ grade**	**Screening measure**	**Purpose of study**	**Finding (Difference in general cognitive function between groups)**	**Screening cut-off**
Zheng et al. (41)	Beijing, China	*n =* 180 CHF 83.9 ± 5.4 years old	NYHA II-III	MoCA	To investigate the prevalence of CI in the elderly patients with CHF, and to describe the clinical characteristics	75.6% of elderly CHF patients had CI. They were characterized by female, poor cardiac function, high glycemic level, low education level, low hemoglobin level and LVEF	26
Xianbin et al. (42)	Guizhou, China	n = 43 CHF 63.73 ± 6.88 years old *n* = 36 non CHF 62.96 ± 7.31 years old	NYHA II-IV LVEF ≤ 50%	MMSE	To explore the changes of cognitive function in patients with CHF	The prevalence of CI in CHF group was 79.07%. The total scores of MMSE and its subtests in visual spatial ability, language ability, attention and working memory, memory and orientation in CHF group were significantly lower than those without CHF.	24
Yunling et al. (37)	Kunming, China	*n =* 98 CHF	LVEF <45%	MMSE	To investigate the factors associated with CI in elderly patients with CHF	Patients with CHF had a higher prevalence (25–50%) of CI, which was associated with older age, no formal education, and decreased LVEF	27
Siqi et al. (52)	China	*n =* 990 HF >18 years old	NYHA	MoCA	To explore the prevalence of CI in Chinese HF patients and its impact on prognosis	63.4% of HF patients had CI. MoCA <26 was an independent risk factor for all-cause death, cardiovascular related death and major cardiovascular and cerebrovascular events in patients with HF.	26
Xiaolin et al. (43)	Shihezi, China,	*n =* 100 CHF 70.39 ± 7.21 years old *n =* 100 non CHF 69.68 ± 6.13 years old	NYHA II-IV LVEF <50%	MoCA	To explore the correlation between CHF and MCI in the elderly	The prevalence of MCI in elderly patients is 60%. MoCA subtests scores in visual spatial and executive ability, naming, attention, language, abstraction, delayed recall and orientation were significantly lower in those with MCI. Higher NYHA class, low LVEF level, longer duration of HF, and high NT-proBNP levels are associated with MCI.	24
Yang et al. (44)	Kunming, China	*n =* 53 CHF 78 ± 7 years old *n =* 53 CVD without CHF 76 ± 7 years old *n =* 21 control group 77 ± 7 years old	NYHA II-IV LVEF ≤ 50%	MoCA	To explore the cognitive function of patients with CHF	The prevalence of CI in CHF patients is 77.4%. The cognitive impairment is mainly driven by MoCA subtest domains, i.e., poorer visual spatial and executive function, attention, language, and memory. The higher the NYHA class and the lower LVEF level, the more sever the cognitive impairment.	26
Jie et al. (45)	Jiangsu, China	*n =* 55 CHF 81.2 ± 6.7 years old *n =* 50 non CHF 79.7 ± 6.4 years old	NYHA	MoCA	To evaluate the relationship between CHF and CI in the elderly participants	The prevalence of CI in HF group is higher than that in non-HF group (69.1 vs. 49.0%). The cognitive function of HF group was poorer, mainly driven by MoCA subtest domains of visual spatial and executive function, attention and working memory, language and delayed recall.	26
Huifeng et al. (38)	Tianjin, China	*n* = 152 CHF 65.38 ± 10.6 years old	NYHA II-IV	MoCA	Examine the relationship between cognitive function and quality of life in patients with CHF.	CI in patients with HF is mainly due to MoCA subtest domains of language, naming, attention, orientation, abstraction, visual spatial and executive function. There was a negative correlation between cognitive function and quality of life in patients with HF.	26
Xiaojia et al. (39)	Beijing, China	*n* = 267 CHF 63.8 ± 9. 4 years old	NYHA II-IV	MoCA	To explore the cognitive function status and associated factors in hospitalized patients with CHF	37.8% of hospitalized patients with CHF had CI. Older age, low LVEF level, medication non-compliance and poor social support were factors associated with CI.	26
Lianru et al. (50)	Jilin, China	*n =* 76 HF 71 (64-78) years old *n =* 30 non-HF 71 (70-75) years old	NYHA II-IV LVEF > 40%	MMSE	To explore the prevalence and possible risk factors of CI in patients with chronic non-HFrEF.	Patients with chronic non-HFrEF were more likely to develop CI than patients without HF. Low EF, higher NYHA class, high homocysteine level, older age and long history of atrial fibrillation were independent risk factors.	24
Haizhen et al. (49)	Shanxi, China	*n =* 116 HF 68.34 ± 7.22 years old *n =* 120 non HF 69.08 ± 8.41 years old	NYHA	MoCA	To study the correlation between CHF patients and MCI	The MoCA scores of elderly patients with CHF is 21.15 ± 4.22. The educational level of CHF patients was positively correlated with the total score of MoCA. Age, course of disease, cardiac function (NYHA class), levels of ST2 and NT-proBNP in patients with CHF were negatively correlated with the total scores of MoCA.	26
Zhengbo et al. (48)	Chongqing, China	*n =* 98 CHF 71.00 ± 13.00 years old *n =* 98 non CHF 71.00 ± 14.00 years old	NYHA II-IV	MoCA	To study the correlation between CHF and CI.	The prevalence of CI in CHF patients is 67.35%. The scores of MoCA subtest domains such as spatial executive ability and delayed recall were poorer in cognitive impaired patients with HF. The decline of cognitive function in patients with HF affected the quality of life in varying degrees, especially in physical strength, social and emotional functioning.	26
Zhengbo et al. (47)	Chongqing, China	*n =* 150 CHF 69.4 ± 13.3 years old *n =* 142 non CHF 69.7 ± 9.7 years old	NYHA I-IV	MoCA	To study the correlation between CHF and CI, and the effect of CI on the quality of life of patients with CHF.	The prevalence of CI in patients with CHF is 66%. The physical, social, and emotional issues, and poorer quality of life in patients with CHF complicated with CI were more than those in patients without CI.	26
Hongbin et al. (40)	Shenyang, China	*n =* 222 60.64 ± 15.18 years old	NYHA II-IV	MMSE	To study the factors associated with CI in patients with CHF.	Age and NYHA class were negatively correlated with MMSE scores. LVEF and years of education were positively correlated with MMSE scores.	24
Xiaoli et al. (46)	Shenyang, China	*n =* 80 CHF 70.2 ± 7.6 years old *n =* 50 non CHF 69.3 ± 8.5 years old	NYHA III-IV	MoCA	To examine the prevalence of CI in CHF and the impact of blood pressure on CI.	58.8% of CHF patients have CI. CI is mainly due to MoCA subtest domains such as visual spatial and executive function, attention, language, abstraction and delayed recall. Coronary heart disease, hypertension, diabetes, COPD, SBP and DBP were associated with CI in HF.	26
Lee et al. (36)	Korea	*n =* 132 HF 60 (12.8) years old	NYHA I-IV	MoCA	To explore factors associated with self-care among HF patients with and without MCI.	Social support and executive function subtest scores of the MoCA were positively associated with self-care in HF patients with MCI.	24

### Cognitive Screening Tools

There were 5 studies that applied cognitive screening tools, including the MMSE (19, 34, 35, 51, 52), the MoCA (19, 52), and the Mini-Cog (51) (Table 1). When considering the utility of cognitive tests for screening purposes, discriminant indices of the screening instruments, including sensitivity, specificity, PPV, and NPV, were examined.

The QUASAD-2 and STARD tools were used to evaluate the results of bias and applicability. The aims of the studies did not examine the diagnostic accuracy of cognitive screening, indicating a high risk of quality bias. All the studies adequately presented the sample demographics. One study administered the cognitive screening tool at the same time as the neuropsychological assessment. However, it was unclear as to whether there is blinding between cognitive screening and the reference standard results, representing a source for the potential risk of information bias. Due to different study aims, study populations were heterogeneous with respect to their demographic and clinical characteristics. For example, some studies included patients with HF ≥ 75 years or older only or patients with the first hospitalization. This indicates a high risk of selection bias across the studies.

Three out of the 5 studies (19, 34, 51) were longitudinal studies. One study found that the MMSE and the MoCA were similar in detecting CI [the area under the curve (AUC): 0.74/0.77] (19). However, the authors indicated 74% of patients with CI that would be undetected without formal neuropsychological evaluation when using published the MMSE cutoff value (<24) (54), suggesting that the prevalence of undiagnosed CI in Asian patients with HF is high. Moreover, N-terminal pro-brain natriuretic peptide (NT-proBNP) was associated with CI. Thus, the MMSE <28 and the MoCA <25 were recommended as the optimal cutoff values (sensitivity: 0.79/0.71, specificity: 0.63/0.61) and a high NT-proBNP level might be considered a high risk for CI and require formal evaluation. Another longitudinal study compared the prognostic ability of two cognitive screening tools (the Mini-Cog and the MMSE) for older patients with HF (51). The authors reported that the Mini-Cog could predict all-cause death better than the MMSE in terms of the AUCs (0.59 vs. 0.52); however, there was no significant statistical difference between these two tests. The Mini-Cog takes less time in test administration than the MMSE, thus the Mini-Cog was suggested for elderly patients with HF. The third study examined HF-related hospital readmission, all-cause mortality within 2 years after discharge, and the prognostic value of the MMSE (34). The results showed that even a slight decline in cognitive function measured by the MMSE (cutoff point <28, sensitivity: 71%, specificity: 41%) could lead to an increased risk of death or readmission in patients with HF.

The remaining 2 studies were cross-sectional studies on the subjective and objective evaluation of cognition. One study (35) examined the degree to which subjective and objective evaluations of cognition coincide and suggested an objective cognitive screening tool that is required for patients with HF. The second study (53) compared the MoCA (Beijing) and the MMSE as screening tools for patients with HF. The MoCA was found to be a more sensitive tool to detect CI than the MMSE when the MoCA <26 (sensitivity: 90 vs. 18%, specificity: 87 vs. 100%).

### Measure of Global Cognitive Function

A total of 16 studies used brief cognitive screening tools as a measure of global cognition. These brief cognitive tools included the MMSE and the MoCA (Table 2). A total of 13 publications (37, 39, 41–50, 52) indicated that the prevalence of CI in patients with HF is often higher than in patients without HF. A total of 7 studies (38, 42–46, 48). showed that the cognitive function of patients with HF had poorer visual, spatial, and executive abilities, namely, attention, language, orientation, working memory, abstraction, memory, and delayed recall. A total of 8 studies (37, 39–41, 43, 44, 49, 50) examined possible risk factors of CI in patients with HF and indicated that educational level, left ventricular ejection fraction (LVEF), medication compliance, and social support in patients with HF were positively correlated with cognitive function, while age, disease duration, the New York Heart Association (NYHA) class, suppression of tumorigenicity 2 (ST2), and NT-proBNP levels were negatively correlated with cognitive function. Among them, 2 studies (47, 48) showed that the decline of cognitive function in patients with HF affected the quality of life to varying degrees, especially in the physical strength, social, and emotional functioning. A total of 1 study (52) reported that CI is an independent risk factor for all-cause death, cardiovascular death, and major cardiovascular and cerebrovascular events in patients with HF.

## Discussion

This systematic review examined the utility of cognitive screening tests in Asian patients with HF from June 1984 to June 2020. Brief cognitive tests have been widely used as a screening tool to detect CI or a measure of global cognitive function in patients with HF. These tests included the MMSE, the MoCA, and the Mini-Cog. In Asian studies, we found that the MMSE and the MoCA are widely used. Six of the 21 studies in this review used the MMSE to identify CI. A total of 3 studies used the MMSE in conjunction with other screening measures. The MMSE is one of the most influential and popular cognitive screening tools in the world, which is used to evaluate the dysfunction of multiple cognitive fields. It has the characteristics of clinical operability. However, the items contained in the MMSE are too simple to reflect the attention, language fluency, and abstract thinking of patients with HF. In our review, 12 studies used the MoCA to screen CI of patients with HF and 2 studies used the MoCA with other cognitive screening tools. The MoCA has a short test administration time and high sensitivity, which is suitable for cognitive screening. However, there are some problems with its application in Asian countries. The MoCA has many different versions and words with western characteristics (such as a church), which restrict its promotion and application. Only 1 study used the Mini-Cog, which showed that the Mini-Cog is rarely used in Asian countries. As a quick and simple cognitive screening tool, the Mini-Cog is objective. Its scores are not easily affected by different language and education levels. However, the research on its reliability and validity is still controversial and needs further discussion in the future. In addition, the western countries have many other cognitive screening tools, such as the National Institute for Neurological Disorders and Stroke-Canadian Stroke Network (NINDS-CSN) 5-Min screen, the modified MMSE, cognitive assessment battery, and consortium to establish a registry for Alzheimer's disease. Unfortunately, the above tools are rarely utilized in Asian countries. In the future, we should further evaluate the diagnostic accuracy of the above cognitive screening tools to select more suitable tools for patients with HF.

Most studies used the published cutoffs of the MoCA <26 and the MMSE <24 to qualify as CI. However, the MoCA <26 was initially applied to screen elderly patients with MCI (55), while the MMSE cutoff <24 was designed as a practical method for assessing cognitive state and detecting dementia within a psychiatric setting (54). Therefore, the original cutoff values of the MMSE and the MoCA may not be suitable for cognitive screening in Asian patients with HF. Dong (19) and their colleagues established the MMSE <28 and the MoCA <25 with acceptable sensitivity (0.79/0.71) and NPV (0.79/0.73), however suboptimal specificity (0.63/0.61) and PPV (0.62/0.59). Such the MMSE and the MoCA cutoff points differ from the published cutoffs (the MMSE <24, the MoCA <26), but are established among the Asian HF population and, thus, more suitable. The MMSE <28 and the MoCA <25 cutoff values are consistent with the findings from studies of Hawkins et al. (56) and Ciesielska et al. (57) based on the western population, which indicated the cutoff scores need to be HF population specific. The above findings provide some preliminary support for the use of the MMSE and the MoCA as cognitive screening tools for the HF population. However, these findings should be considered in the context of some key methodological issues. First, among the 5 studies that reported cognitive screening tools, 3 studies failed to report sensitivity and specificity. Second, the differing MoCA cutoff points generated from various studies make recommendations for clinical practice difficult. For example, Dong et al. showed that the optimal cutoff points of the MoCA <25, yet, Davis et al. indicated that the MoCA <26 can identify 54% of participants with MCI, while the MoCA <22 can detect 17% of participants with MCI (22). Finally, fewer studies adjusted the cutoff points of cognitive screening tools according to the demographic (e.g., age, education, premorbid intelligence) and HF variables (e.g., the NYHA classification). Thus, it is possible that unadjusted cutoff points of these cognitive screening measures for the HF population have suboptimal sensitivity and specificity. In view of the significant impact of demographic factors (e.g., age) on cognition, future studies should establish age- and education-adjusted cutoff points.

With regard to general study methodological issues, previous studies did not take into account cognitive screening at different time points of HF trajectory. Cognitive function may change significantly during the course of HF trajectory and screening cutoff points established from early phase/newly diagnosed HF may not represent cutoff points for patients with chronic HF with years of disease duration. Therefore, we recommend cognitive screening and gold standard neuropsychological assessments to be conducted as time congruent as possible. Moreover, due to the lack of PPV and NPV in many studies, we were unable to compare these discriminant indices, which take into account the prevalence of CI in various HF populations. In addition, it is crucial to choose culturally and linguistically appropriate cognitive screening tools for the different populations in various countries. Most cognitive screening tools were initially developed as English language-based tests, with some being translated and validated in a limited subset of non-English languages (e.g., Chinese, Japanese, Korean) (40).

In a sample of 100 patients with HF, 44% were identified as having CI that would otherwise not have been identified (19). Furthermore, CI could affect self-care because of medication complexity, numerous lifestyle changes, and recognition of HF symptoms (58). Therefore, even though subtle CI could render patients with HF vulnerable to adverse health outcomes, including poor medication compliance and daily functioning, thus increasing healthcare burden and resource utilization. Future studies should increase the sample size and select appropriate cognitive screening tools for patients with HF according to different demographic and clinical characteristics of patients with HF (e.g., age, education level, the NYHA classification). In doing so, consensus and evidence-based guidelines could be developed with recommendations on how we screen for CI to customize the management of cognitively impaired patients with HF (23).

### Strengths and Limitations of the Study

The previous reviews focused on the western HF population, but Asia has a higher prevalence of HF, as well as a higher prevalence of CI in these patients [number of patients with HF: western countries (e.g., USA has 5 million) (7) vs. Asian countries (e.g., China has 13.7 million) (4); CI prevalence in HF: western countries (e.g., USA 10–15%) (23) vs. Asian countries (e.g., Singapore 44%)] (19). In view of more prevalent HF and CI issues, it is novel and strength for our review to focus on the Asian HF population, so as to provide the evidence for population-specific cognitive screening practice.

In the Asian region, in addition to English databases, we have searched major Chinese databases This is due to a large HF population reported in China, i.e., an estimate of 13.7 million patients with HF of Chinese people aged ≥ 35 years old (5), which is almost half of the estimated global HF population.

Our rigorous screening of the literature published over a longer period (>30 years) and using a quality appraisal approach is also a strength. Two independent reviewers screened and extracted relevant studies and conducted a quality appraisal to prevent selection bias and ensure accuracy. Any disagreements have been resolved by a third reviewer.

The limitations of our review are as follows. First, we only searched for studies that were published in Chinese and English. We did not search for studies that were published in other Asian languages such as Hindi, Indonesian, Bengali, and Japanese, which would lead to publication bias. Second, in our review, literatures that meet the inclusion criteria are limited. Third, we cannot conduct meta-analysis because of a small sample and a high risk of bias. This is a barrier for us to evaluate and compare the quality of brief cognitive tests as screening measures across studies. Fourth, most studies did not conduct formal neuropsychological assessments and were lacking in data on the sensitivity and specificity of cognitive screening tools. Hence, we cannot calculate the optimal cutoff value.

## Conclusion

In conclusion, brief cognitive tests have been used to screen Asian patients with HF for CI or as a measure of global cognitive function. However, the review studies did not adequately validate cognitive screening measures against a gold standard neuropsychological assessment in line with STARD criteria. Future studies need to address methodological issues mentioned in this review, so as to validate cognitive screening measures in a larger sample of Asian patients with HF following STARD and QUADAS-2 criteria.

## Data Availability Statement

The raw data supporting the conclusions of this article will be made available by the authors, without undue reservation.

## Author Contributions

QN designed this study and drafted the manuscript with the help of YD. QN and LT screened and assessed included literatures. WL and FW reviewed the manuscript. YD conceptualized this study, contributed to the design, and provided a critical review of the manuscript. All authors contributed to the article and approved the submitted version.

## Funding

This study was supported by the Research Program of Humanities and Social Sciences in Colleges and Universities of the Department of Education of Shandong province (No. J18RB063). YD is supported by the Singapore Medical Research Council (NMRC) Transition Award (NMRC/TA/0060/2017).

## Conflict of Interest

The authors declare that the research was conducted in the absence of any commercial or financial relationships that could be construed as a potential conflict of interest.

## Publisher's Note

All claims expressed in this article are solely those of the authors and do not necessarily represent those of their affiliated organizations, or those of the publisher, the editors and the reviewers. Any product that may be evaluated in this article, or claim that may be made by its manufacturer, is not guaranteed or endorsed by the publisher.
